# A cytosine modification mechanism revealed by the structure of a ternary complex of deoxycytidylate hydroxymethylase from bacteriophage T4 with its cofactor and substrate

**DOI:** 10.1107/S2052252518018274

**Published:** 2019-01-24

**Authors:** Si Hoon Park, Se Won Suh, Hyun Kyu Song

**Affiliations:** a Department of Life Sciences, Korea University, 145 Anam-ro, Seongbuk-gu, Seoul 02841, Republic of Korea; b Departments of Chemistry, Seoul National University, Kwanak-ro 1, Kwanak-gu, Seoul 08826, Republic of Korea

**Keywords:** bacteriophage T4, cytosine modification, deoxycytidylate hydroxy­methylase, thymidylate synthase

## Abstract

To protect viral DNA against the host bacterial restriction system, bacterio­phages utilize a special modification system – hydroxymethylation – in which dCMP hydroxymethylase (dCH) converts dCMP to 5-hydroxymethyl-dCMP (5hm-dCMP) using *N*5,*N*10-methylenetetrahydrofolate as a cofactor. Despite shared similarity with thymidylate synthase (TS), dCH catalyzes hydroxylation through an exocyclic methylene intermediate during the last step, which is different from the hydride transfer that occurs with TS. In contrast to the extensively studied TS, the hydroxymethylation mechanism of a cytosine base is not well understood due to the lack of a ternary complex structure of dCH in the presence of both its substrate and cofactor. This paper reports the crystal structure of the ternary complex of dCH from bacteriophage T4 (T4dCH) with dCMP and tetrahydrofolate at 1.9 Å resolution. The authors found key residues of T4dCH for accommodating the cofactor without a C-terminal tail, an optimized network of ordered water molecules and a hydrophobic gating mechanism for cofactor regulation. In combination with biochemical data on structure-based mutants, key residues within T4dCH and a substrate water molecule for hydroxymethylation were identified. Based on these results, a complete enzyme mechanism of dCH and signature residues that can identify dCH enzymes within the TS family have been proposed. These findings provide a fundamental basis for understanding the pyrimidine modification system.

## Abstract 2

The structure of dCMP hydroxymethylase from bacteriophage T4 in complex with the substrate dCMP and cofactor THF was determined at 1.9 Å resolution, providing the first view of the ternary complex of a pyrimidine hydroxymethylase.

## 1. Introduction   

Hydroxymethylation of the pyrimidine bases of DNA is associated with diverse cellular processes including epigenetic gene regulation, embryonic development and host immune escape (Weigele & Raleigh, 2016 ▸; Gommers-Ampt & Borst, 1995 ▸; Pastor *et al.*, 2013 ▸; Borst & Sabatini, 2008 ▸; Warren, 1980 ▸; Olinski *et al.*, 2016 ▸). 5-Hydroxymethylcytosine (5hmC) was first identified in T-even phages (Wyatt & Cohen, 1952 ▸) and was found to protect the viral genome from the restriction-modification system of the bacterial host (Labrie *et al.*, 2010 ▸; Samson *et al.*, 2013 ▸). To generate a modified cytosine in T4 bacteriophage, a multi-enzyme cascade is involved (Miller *et al.*, 2003 ▸; Warren, 1980 ▸). First, T4 deoxycytidine triphos­phatase cleaves the phosphodiester bond of dCTP to produce dCMP. Next, dCMP is converted to 5hm-dCMP by dCMP hydroxymethylase (dCH) using *N*5,*N*10-methylenetetra­hydrofolate (mTHF). Then, 5hm-dCMP is phosphorylated to 5hm-dCTP by T4 deoxynucleoside monophosphate kinase from T4 bacteriophage and nucleoside diphosphate kinase from the host bacteria. Finally, T4 DNA polymerase incorporates the modified base at the position of the cytosine within the phage genome. The substituted 5hmCs are further glycos­ylated by α/β glucosyltransferase. 5hmC is involved in nucleotide modification and also in nucleoside antibiotic synthesis (Cooper *et al.*, 2014 ▸; Larsen *et al.*, 1989 ▸; Feduchi *et al.*, 1985 ▸). For example, 5hmC is a precursor for mildiomycin, a nucleoside antibiotic (Li *et al.*, 2008 ▸; Zhao *et al.*, 2016 ▸) whose synthesis is catalyzed by CMP hydroxymethylase (CH) MilA from *Streptomyces rimofaciens* ZJU5119 (SrMilA).

In *Bacillus subtilis* bacteriophage SPO1, a similar pyrimidine modification system called uracil hydroxymethylation also exists (Wilhelm & Rüger, 1992 ▸). The 5-hydroxymethyl dUMP is catalyzed by deoxyuridylate hydroxymethylase and replaces thymine bases in the viral genome to escape specific recognition and cleavage from type-II restriction endo­nucleases (Berkner & Folk, 1977 ▸; Huang *et al.*, 1982 ▸). In mammals, 5hmC is found in embryonic stem cells and Purkinje neurons, and is generated from 5-methylcytosine by the catalytic action of ten–eleven translocation (TET)-family proteins, which are mammalian homologs of the J-binding protein (JBP) 1/2 (Tahiliani *et al.*, 2009 ▸; Kriaucionis & Heintz, 2009 ▸). In unicellular eukaryotes such as kinetoplastids – which include *Trypanosoma* and *Leishmania – *β-d-glucosyl hydroxymethyluracil (base J) is present in telomeric repeats and is formed via two steps, hydroxylation of thymidine by JBP 1/2 [Fe(II) and 2-oxoglutarate-dependent dioxygenases] and glucosylation by an as-yet unidentified glucosyltransferase (van Leeuwen *et al.*, 1998 ▸; Yu *et al.*, 2007 ▸). The exact role of base J remains unclear; however, it is thought to be associated with gene repression and telomeric function (Borst & Sabatini, 2008 ▸).

The dCH from T4 bacteriophage (T4dCH) was the first identified viral enzyme, which catalyzes the conversion of dCMP to 5hm-dCMP using mTHF and water as methyl and hydroxyl donors, respectively (Cohen, 1994 ▸; Hardy *et al.*, 1995 ▸). Despite its low sequence identity, dCH belongs to the thymidylate synthase (TS) superfamily due to its similar tertiary structure (Finer-Moore *et al.*, 2003 ▸; Song *et al.*, 1999 ▸). The enzyme characteristics of TS have been well studied for *de novo* synthesis of DNA by supplying thymidine bases (Carreras & Santi, 1995 ▸; Finer-Moore *et al.*, 2003 ▸; Stroud & Finer-Moore, 2003 ▸). The reaction steps of dCH are similar to those of TS; in particular, cofactor protonation and formation of the exocyclic methylene intermediate have been similarly observed in the enzyme reaction steps (Butler *et al.*, 1994 ▸; Hardy *et al.*, 1995 ▸). Nevertheless, there are distinguishable differences between dCH and TS. The first is the structural difference of their cofactor binding sites. In the ternary complex of TS, the C-terminal region plays a critical role in occluding the bound THF from the solvent environment, although this may not hold as it is unclear whether this would occur in dCH, since it lacks the C-terminal tail required for THF recognition. The second difference occurs in the enzyme reaction steps. TS produces thymine as a methylated form of uracil, but dCH hydroxymethylates dCMP. Thus, dCH proceeds through another reaction step for hydroxylation to occur rather than hydride transfer; this mechanism is not yet well understood. Owing to the difference in the last reaction step – even though dCH and TS both use mTHF as a cofactor – the reaction products of the cofactor for dCH and TS are tetrahydrofolate (THF) and dihydrofolate (DHF), respectively. For cofactor recycling, serine hydroxymethyltransferase converts THF to mTHF. However, for TS, dihydrofolate reductase (DHFR) is required for methyl-group shuttling. This is due to evolutionary differences as dCH is only found in several bacterial viruses, whereas TS is the sole *de novo* enzyme that is universally conserved among all organisms.

We previously solved the apo and binary dCMP-complexed structures of T4dCH (Sohn *et al.*, 1999 ▸). These atomic-resolution structures provided a wealth of information on the substrate specificity and catalysis of dCH as a prototype pyrimidine hydroxymethylase. It has been hypothesized that the reaction requires a water transfer to the exocyclic methylene group of cytosine. However, due to the lack of a dCH–dCMP–THF ternary complex structure, our understanding of the mechanism has remained very limited. Here, we report the crystal structure of the ternary complex of T4dCH (C148S/D179N mutant) bound to dCMP and THF at 1.9 Å resolution. The structure reveals how dCH recognizes THF despite the lack of a C-terminal tail, which is a critical region in TS (Variath *et al.*, 2000 ▸). Comparing the ternary complex of dCH with that of TS from *E. coli* (EcTS), a clear difference in THF geometry upon complex formation was identified. This ternary complex structure allowed us to design several mutants in order to understand the second enzyme step: hydroxylation. Furthermore, we measured dCMP binding and hydroxymethylation activities to further enhance our understanding of the hydroxylation reaction mechanism of dCH, which has been a long-standing question in the field of pyrimidine hydroxymethylation.

## 2. Materials and methods   

### 2.1. Cloning, protein expression and purification   

The *T4dCH* gene was amplified by PCR using a gBlock synthetic gene fragment (Integrated DNA Technologies, Inc.). The amplified products were cloned into a modified pET vector, which added a hexahistidine tag to the C-terminus. For crystallization, substrate binding and the activity assay, we introduced mutations into the cloned wild-type (WT) *T4dCH* gene using PCR-based, site-directed mutagenesis. Purification of T4dCH has been described in a previous report (Sohn *et al.*, 1999 ▸). In brief, the WT and mutant constructs were expressed in BL21(DE3) cells at 310 K with the addition of 0.5 m*M* IPTG for 3 h. Cells were resuspended in lysis buffer [50 m*M* Tris–HCl pH 8.0, 150 m*M* NaCl and 1 m*M* Tris-(2-carboxy­ethyl)phosphine hydrochloride (TCEP)] and lysed by ultrasonication. The cell lysate was clarified by centrifugation and loaded onto a HisTrap FF column (GE Healthcare) for affinity purification. Finally, the fraction containing T4dCH was loaded onto a HiLoad 16/600 Superdex 200 pg column (GE Healthcare) for size-exclusion chromatography. Highly pure WT and mutant constructs of T4dCH were used for subsequent crystallization, binding studies and the activity assay.

### 2.2. Crystallization   

Crystallization was performed using the hanging-drop vapor-diffusion method with 24-well plates at 298 K. The crystallization conditions were previously described by Sohn *et al.* (1999 ▸); however, a new tetragonal crystal form was obtained by adding 10%(*v*/*v*) 1.0 *M* NaI to the crystallization conditions. Structures of T4dCH in the apo (PO_4_-bound) and binary complex (dCMP-bound) states crystallized previously in the monoclinic crystal form showed that the putative THF-binding pocket is too narrow for accommodating the cofactor (Sohn *et al.*, 1999 ▸). To determine the ternary T4dCH–dCMP–THF complex, we attempted to crystallize T4dCH WT in the presence of dCMP and THF. The stock solutions of 1.0 *M* dCMP and 100 m*M* THF were prepared by dissolving them in distilled water and adjusting to pH 7.0 with 10 *N* NaOH. Interestingly, the molecular weight of dimeric T4dCH increased slightly with the addition of THF (see Fig. S1 of the supporting information). Furthermore, the transparent protein crystals turned light yellow by soaking or co-crystallizing with THF (Fig. S2). Unfortunately, we could not obtain any reliable electron density for THF in the putative binding site. Therefore, we introduced active-site mutations, C148S/D179N, to stabilize the intermediate state. The C148S/D179N double mutant was crystallized after pre-incubation overnight with 30 m*M* dCMP and 1 m*M* THF at 277 K. The ternary complex crystals were grown in 100 m*M* Tris–HCl pH 8.5 and 0.96 *M* sodium citrate [Fig. S2(*c*)]. In the preliminary X-ray experiments using a home source, the electron-density map of THF was weak and, to improve the occupancy of THF, the crystals were further soaked with 25 m*M* THF at 277 K for 12 h before data collection.

### 2.3. Data collection, structure determination and refinement   

For the X-ray crystallography experiments, crystals were transferred to a reservoir solution containing 25%(*v*/*v*) glycerol before flash-cooling in liquid nitrogen. X-ray data were collected from a single crystal at 100 K using a laboratory X-ray diffractometer or the BL-5 C synchrotron at the Pohang Light Source (PLS) Facility in South Korea (Park *et al.*, 2017 ▸). The diffraction data were processed using *HKL-2000* (Otwinowski & Minor, 1997 ▸). We obtained the initial phases of the tetragonal crystal of apo T4dCH WT using two different methods. The first was molecular replacement (MR) using *Phaser* (McCoy *et al.*, 2007 ▸) and the previously solved T4dCH structure as a search model (PDB entry: 1b5e). The second was the single-wavelength anomalous dispersion (SAD) method using data collected with the Cu *K*α laboratory source. Bound iodide was successfully found as an anomalous scatterer using *Autosol* (Terwilliger *et al.*, 2009 ▸) and played a critical role in the tetragonal crystallographic packing (Fig. S3). The ternary complex data were collected at PLS, and the phases were obtained by MR with the refined apo model of the tetragonal crystal form. For modeling ligands, we used the *eLBOW* module in *PHENIX* together with ligand geometric constraints (Moriarty *et al.*, 2009 ▸). Bound dCMP was clearly visible in the initial *F*_o_ − *F*_c_ difference Fourier map contoured at 3.0σ, and extra electron density around the pyrimidine base of dCMP was also observed. During several refinement cycles of the model containing dCMP and iodide, the difference map for THF became clearer and was contoured at 3.0σ. To fit the THF molecule into the electron-density map, we used the *LigandFit* module in *PHENIX* and carefully optimized the positions of the THF atoms manually using *Coot* (Emsley & Cowtan, 2004 ▸; Venkatachalam *et al.*, 2003 ▸). The final refinement and validation of all models were obtained using *PHENIX* (Zwart *et al.*, 2008 ▸). The data collection, phasing and refinement statistics are summarized in Table 1 ▸. For interface analysis of ligand–protein contacts, the *PISA* server in the *CCP*4 suite and *LIGPLOT* were used (Krissinel & Henrick, 2007 ▸; Wallace *et al.*, 1995 ▸). All structural figures were prepared using *PyMOL* (Schrödinger LLC).

### 2.4. Thermal-shift assay   

The thermal-shift assay (TSA) was performed using a LightCycler 480 II real-time PCR instrument (Roche Diagnostics). The experiment was conducted using a TSA buffer (50 m*M* HEPES pH 7.5, 300 m*M* NaCl and 1 m*M* TCEP). A solution of total volume 20 µl, containing 0.1 mg ml^−1^ of protein, various concentrations of dCMP and 5× Sypro Orange (Life technologies), was placed into the wells of 384-well plates (Roche Diagnostics). The plates were sealed, and the samples were gradually heat-denatured from 298 to 358 K at a rate of 0.5 K min^−1^. Thermal denaturing curves were monitored by the change in fluorescence of Sypro Orange. The fluorescence of the TSA buffer alone was used as a control. A calculation of the *T*_m_ values was performed using the *LightCycler* 480 data analysis software (Roche) that was included with the instrument.

### 2.5. Isothermal titration calorimetry   

All isothermal titration calorimetry (ITC) experiments were performed at 298 K on high-feedback mode with a stirring speed of 750 rev min^−1^ using a MicroCal PEAQ-ITC (Malvern Instruments, Inc.). To measure the affinity of dCMP binding to T4dCH WT and its mutants, we used both ITC1 (50 m*M* HEPES–KOH pH 7.5, 150 m*M* KCl and 1 m*M* TCEP) and ITC2 (50 m*M* HEPES–KOH pH 7.5, 300 m*M* KCl and 1 m*M* TCEP) buffers, since some mutants (S94A and D145N) existed as mixed species of dimer and monomer at high protein concentrations and in low salt. dCMP (1–2 m*M*) was dissolved in matched ITC buffer and injected 19 times (2 µl each) into 200 µl of 80–100 µ*M* T4dCH proteins. All data were analyzed and represented using PEAQ-ITC.

### 2.6. Enzymatic activity assay   

To measure the activity of T4dCH constructs, mTHF was prepared by dissolving THF (Sigma) in 25 m*M* formaldehyde and 10 m*M* KH_2_PO_4_–NaOH (pH 7.5). The assay was performed at 303 K for 1 h in a total volume of 500 µl, containing 100 m*M* HEPES–NaOH (pH 7.5), 50 m*M* 2-mercaptoethanol, 0.4 m*M* mTHF, 0.4 m*M* dCMP and 50 µg of WT or mutant T4dCH. Reactions were quenched by heating for 5 min and then subsequent dilutions to 1.5 ml with a running buffer [3%(*v*/*v*) MeOH and 0.1%(*v*/*v*) formic acid]. The products were resolved by C18 reverse-phase HPLC (Hypersil BDS, 5 µm, 4.6 × 125 mm; Thermo Fisher Scientific) using an FPLC system (GE Healthcare) with the running buffer as the mobile phase. Before injection, all samples were centrifuged at 12 000 rev min^−1^ for 10 min to remove precip­itants. The injection volume was 10 µl with a constant flow rate of 0.3 ml min^−1^. The spectra were monitored at 268 nm. For mass spectroscopy analysis, spectra were obtained using a UPLC system (ACQUITY UPLC; Waters) and a SYNAPT G2-Si high-definition mass spectrometer (Waters) equipped with an electrospray ionization source. Chromatographic separation was performed on an ACQUITY UPLC BEH C18 column (1.7 µm, 2.1 × 100 mm; Waters) using mobile phase *A* [0.1%(*v*/*v*) formic acid in distilled water] and mobile phase *B* [0.1%(*v*/*v*) formic acid in acetonitrile] at a constant flow rate of 0.3 ml min^−1^. The column temperature was set to 313 K. The sample injection volume was 1 µl before 4× dilution using methanol. Chromatograms were detected at 268 nm. The percentage of *B* buffer at time *t* (min) varied according to following scheme: (*t*, *B*), (0, 1), (6.0, 1), (6.2, 100), (7.8, 100), (8.0, 1), (10.0, 1). Mass data acquisition was performed in negative mode. The capillary voltage was 2 kV, and the cone voltage was 30 V. The source temperature was 373 K. The desolvation gas flow was set to 800 l h^−1^, and the desolvation temperature was 623 K. The operating mass range was from 100 to 700 Da.

### 2.7. Size-exclusion chromatography with multi-angle light scattering   

Size-exclusion chromatography with multi-angle light scattering (SEC-MALS) experiments were performed using an FPLC system (GE Healthcare) connected to a MiniDAWN TREOS MALS (Wyatt) and an Optilab rEX differential refractometer (Wyatt). A Superdex200 10/300 GL (GE Healthcare) gel-filtration column was equilibrated with a SEC-MALS buffer [50 m*M* Tris–HCl (pH 7.5), 150 m*M* NaCl and 1 m*M* TCEP]. Bovine serum albumin was used as the isotropic scatterer for detector normalization. Data were evaluated using the Zimm model to fit static light scattering data and drawn in *EASI Graph* using the UV peak information from *ASTRA V* (Wyatt).

## 3. Results   

### 3.1. Structure of the ternary T4dCH–dCMP–THF complex   

**Figure 1:**
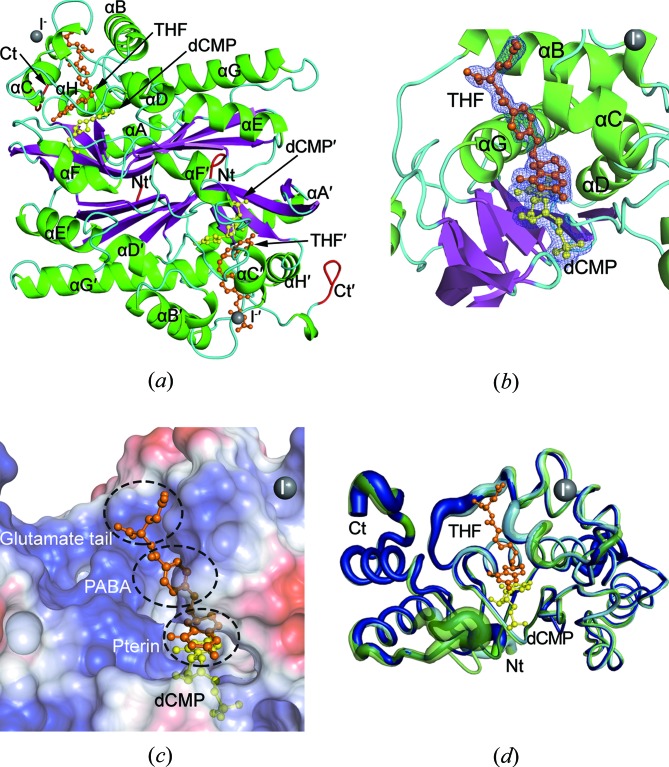
Ternary complex structure of T4dCH with dCMP and THF. (*a*) Ribbon diagram showing the ternary complex structure of dimeric T4dCH viewed along the crystallographic twofold symmetry axis. N- and C-terminal ends are colored red and labeled as Nt and Ct, respectively, Secondary structural elements are colored green (α-helix and 3_10_-helix), purple (β-strand) and cyan (loops). The bound dCMP (yellow) and THF (orange) are drawn using the ball and stick model. The bound iodide is also shown as a gray ball. (*b*) Electron-density maps showing the bound dCMP (yellow) and THF (orange) in the active site of T4dCH. The 2*F*_o_ − *F*_c_ map (blue) is contoured at 1.0σ. (*c*) Ball-and-stick model of dCMP (yellow) and THF (orange) in the deep binding pocket, which is represented as an electrostatic surface. The positively and negatively charged areas are blue and red, respectively. Each subpart of THF is labeled with black dotted circles. (*d*) *B* factor diagram of the apo (green), binary (cyan) and ternary complex (blue) forms. The thickness of the tube reflects the value of the *B* factor, *i.e.* the higher the *B* factor, the thicker the tube.

A reasonable electron density for THF could not be obtained from crystals of T4dCH WT; therefore, the active-site double mutant C148S/D179N was used for crystallization. It has already been reported that Cys148 is a key catalytic residue that acts as a nucleophile and Asp179 is critical for protonation of N3 in the heterocyclic base of dCMP (Graves *et al.*, 1992 ▸; Butler *et al.*, 1994 ▸). Interestingly, T4dCH D179N has a lower *K*_cat_ value – by up to ∼3600-fold – compared with WT, but has little effect on the *K*_M_ values (Graves *et al.*, 1992 ▸). Finally, we crystallized the ternary complex using this inactivated C148S/D179N mutant in space group *I*222 with only one protomer of the T4dCH dimer present in the asymmetric unit. The structure of the ternary T4dCH–dCMP–THF complex was determined at 1.9 Å resolution [Fig. 1 ▸(*a*)]. The final model showed *R*_work_/*R*_free_ values of 0.1565/0.1955, respectively (Table 1 ▸). Both dCMP and THF in the active site were easily built into the electron-density map [Fig. 1 ▸(*b*)]. The THF molecule resides in a narrow and deep binding pocket formed by α-helices B, C and G. The enzymatically active cavity for hydroxymethylation is formed along to the narrow binding site of THF, which links to the dCMP binding site. The THF completely occludes the dCMP molecule from the bulk solvent [Fig. 1 ▸(*c*)]. However, many ordered water molecules are bound to THF, which surrounds dCMP in the active-site cavity.

The Cα root-mean-square deviation (RMSD) between the ternary and binary complex structures is only 0.26 Å, and that between the ternary complex and the apo structure is 0.39 Å. These results suggest that no global conformational changes are triggered by THF binding [Fig. 1 ▸(*d*)]. Moreover, in the active-site cavity, there also appears to be no main-chain movements when both the substrate and cofactor are bound. The Cα RMSD for all matching atoms (residues 5–241) between the ternary and binary complex structures is also only 0.75 Å, and that between the ternary complex and the apo structure is 0.71 Å. The RMSD values for residues within 10 Å of dCMP and THF are also similar, such as 0.78 and 0.79 Å for the binary and apo structures, respectively. When compared with previously reported structures, the current structure shows increased helicity at the N-terminal region, which weakens the hydrogen-bonding interactions of Met1–Glu35′, Ser3–Ile37′ and Ile37–Met1′ (a prime indicates the second protomer in the dimer). The C-terminal region of the current structure is clearly defined in the electron density; however, it was invisible in some of the previously solved structures (PDB entries: 1b49 and 1b5e). Therefore, the N- and C-termini are flexible, as shown by their high *B*-factor values [Fig. 1 ▸(*d*)].

In addition to the substrate and cofactor binding sites in the structure of the ternary complex, we also observe extra globular electron density in the map [Fig. S3(*a*)], which was interpreted as an iodide because a high concentration of sodium iodide was added to the crystallization conditions. Even though only one iodide was found in the asymmetric unit, it was enough to obtain the phases using SAD data that had been collected with the home Cu *K*α X-ray source (Table 1 ▸). The iodide location was close to Val71 (3_10_-helix G1), Thr78 (loop between 3_10_-helix G1 and α-helix C) and Gln83 (α-helix C) of one protomer, and Met111′ and Ala114′ (α-helix E′) of the symmetry mate [Fig. S3(*c*)]. Moreover, iodide binding provides additional crystallographic contacts with the other symmetry mate by hydrogen bonding to the main-chain atoms of Asp73 and Gly76 (α-helix C) and Asn139′ (3_10_-helix G3′) [Fig. S3(*d*)]. This iodide may contribute to the new crystal packing and reduces the *B* factor of α-helix C (Fig. S4).

### 3.2. The THF-binding site   

**Figure 2:**
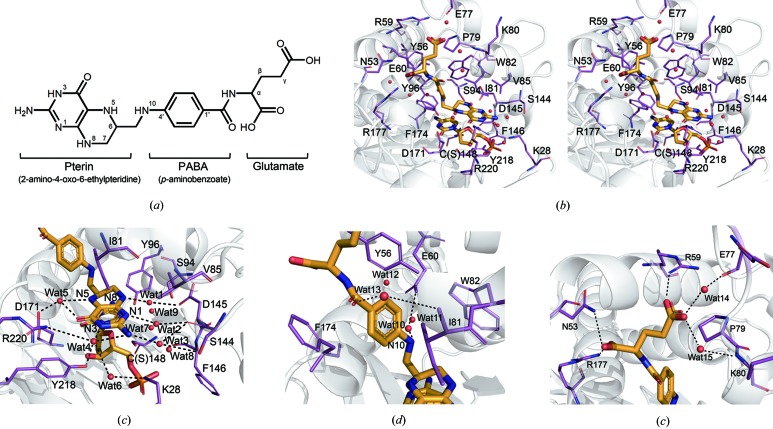
Structure and ligand geometry of THF in the active site of T4dCH. (*a*) Chemical formula of THF. The subparts are named as pterin, PABA and the glutamate tail. The methyl group is linked to the N5 and N10 positions in mTHF. (*b*) Stereo diagram showing the overall interaction of THF in the binding site of T4dCH. THF and dCMP are drawn as stick models (orange). Oxygen and nitrogen atoms are colored red and blue, respectively. The THF-recognizing residues are drawn as thin stick models (purple) with the cartoon representation of T4dCH (off-white). Water molecules are depicted as red balls. (*c*) Interaction of the pterin core in the active site. Hydrogen bonds are represented by dash lines. (*d*) Recognition of the PABA ring and the hydrogen-bonding interaction at the N10 position with Glu60. (*e*) Hydrophilic interaction of the glutamate tail with charged residues on the surface of T4dCH. Ionic and hydrogen interactions are drawn by dashed lines.

THF is composed of three parts: a pterin core, a *para*-aminobenzoic acid (PABA) ring and a glutamate tail [Fig. 2 ▸(*a*)]. The pterin core sits on the ring of the pyrimidine base of dCMP in the active site, and the PABA ring is embedded in the narrow crevice at the THF-binding site. Lastly, the glutamate tail forms contacts with positively charged surface regions around the entrance of the crevice [Fig. 1 ▸(*c*)]. Interestingly, dCH specifically recognizes each part of THF using numerous residues in the binding sites [Fig. 2 ▸(*b*)]. First, the pterin core is recognized indirectly via water molecules, which form a hydrogen-bonded network with the polar atoms of the active site residues. In the same plane of the pterin ring, there are five water molecules (Wat1, Wat2, Wat3, Wat4 and Wat5) that interact with all of the nitrogen atoms (N1, N3, N5, N8 and 2-amino group) of the pterin core [Fig. 2 ▸(*c*)]. These ordered waters are also connected to side chains (Lys28, Ser94, Tyr96, Ser144, Asp145, Asp171, Tyr218 and Arg220) and main chains (Ser144, Phe146 and Cys148) in the active site through hydrogen-bonded networks. This interaction is also contributed by secondary water molecules (Wat6, Wat7, Wat8 and Wat9), which interact with the primary water molecules for indirect stabilization of the pterin core [Fig. 2 ▸(*c*)]. Among the primary and secondary water molecules, three water molecules (Wat1, Wat2 and Wat3) are close to the catalytic nucleophile (Cys148) and the C5 position of dCMP. Another set of three water molecules (Wat1, Wat7 and Wat9) is located close to the expected C7 position of the cytosine ring. Intriguingly, a water (Wat1) is identified at both positions and was not observed in the previous binary complex structure (Sohn *et al.*, 1999 ▸). Therefore, this water might serve as an oxygen atom source for the hydroxylation in the last step of the hydroxymethylation reaction (which is described in more detail later). In addition to hydrogen-bonding interactions, there are also van der Waals interactions. The hydrophobic side chains of Ile81, Val85 and Tyr218 contribute to the stabilization of the planar orientation of the pterin ring [Fig. 2 ▸(*c*)]. Indeed, the main determinant for the positioning of the pterin ring is the substrate dCMP, wherein both the cytosine ring and the ribose moiety are critical for correctly orientating THF during the enzyme reaction [Fig. 2 ▸(*c*)].

Secondly, the PABA ring is recognized in the narrow binding crevice by the aromatic residues Tyr56, Trp82 and Phe174 as contributors of major hydrophobic interactions. The side chain of IIe81 interacts with the benzene ring of PABA on the opposite side [Fig. 2 ▸(*d*)]. Moreover, the main-chain nitrogen atom of Ile81 interacts with the hydroxyl group of PABA via a water-mediated hydrogen bond. The conformation of Ile81 appears to be critical for opening the THF cofactor binding site, which will be described later. The side-chain carboxylate of Glu60 also interacts with the N10 position of the PABA by a water-mediated hydrogen bond. The Glu60 is a highly conserved catalytic residue in all TS/CH/dCH family members (Glu58 in EcTS and Glu68 in SrMilA) and plays a critical role in water-mediated proton­ation of mTHF, which is an important step in methylation. In addition to the aforementioned water (Wat10), Glu60 possesses two more water molecules (Wat11 and Wat12) that might help with immediate recharging of protons during catalysis [Fig. 2 ▸(*d*)].

Lastly, the glutamate tail contacts the positively charged surface directly through ionic interactions. There are two carboxylate moieties on the glutamate tail [Fig. 2 ▸(*a*)]. The one close to Cα is recognized by the side chains of Asn53 and Arg177, whereas the other one interacts in a more complicated manner, such as via a salt bridge (Arg59), water-mediated hydrogen bonds (Wat14 with the main-chain oxygen of Glu77–Wat14, and Wat15 with the main-chain nitrogen of Lys80) and van der Waals interactions (Pro79) [Fig. 2 ▸(*e*)]. Notably, the positively charged surface required for THF recognition was not detected in the apo enzyme or the binary complex of T4dCH with dCMP (Song *et al.*, 1999 ▸), suggesting that the THF cofactor induces rearrangement of the surface charges at the entrance of the binding crevice.

### 3.3. The entrance for THF and adjustment of residues for binding   

The previously solved phosphate- (PDB entry: 1b49) and dCMP-bound T4dCH (PDB entry: 1b5e) structures had extremely narrow holes for the putative THF binding sites (Sohn *et al.*, 1999 ▸). When we compare the ternary complex with the previous structures using the electrostatic potential surface model, the current T4dCH structure possesses a more open conformation of the entrance for accommodating THF in the active site [Figs. S5(*a*), S5(*b*) and S5(*c*)]. The closed conformation of the apo or binary complex blocks the PABA ring-binding region. THF binding induces local structural changes in Ile81 and Phe174 [Figs. S5(*d*) and S5(*e*)]. The branched side chain of Ile81 and the aromatic ring of Phe174 rotate to the outside of the active site, which opens the PABA ring binding site and simultaneously closes the dCMP binding pocket. In addition, the flipped side chain of Val85 stabilizes the pterin ring [Fig. S5(*f*)]. We also found that the surface-charge rearrangements are triggered around the entrance of the active site in the open form [Fig. S5(*c*)]. It is induced by flipping of the side chain of Asn53 [Fig. S5(*g*)]; the positively charged area of the THF binding site becomes enlarged and the glutamate tail easily binds to the entrance of the active site. In the THF binding site, the carboxylate of Glu60 moves approximately 1.0 Å towards a water molecule (Wat10) for recognizing the N10 position of the PABA ring [Figs. S6 and 2 ▸(*d*)]. The side chains of various residues (Asn53, Tyr56, Arg59, Glu60, Ile81, Trp82, Val85, Phe174 and Tyr218) are adjusted for THF recognition by shifting from 0.5 to 1.3 Å (Figs. S5 and S6); although the movement of the main chains within the determined structures is not significant based on the RMSD values [Fig. 1 ▸(*d*)]. Therefore, it would be easier to bind and dissociate the THF in the open conformation by the local side-chain adjustment found in the ternary complex structure of T4dCH.

### 3.4. Comparison of THF recognition with thymidylate synthase   

**Figure 3:**
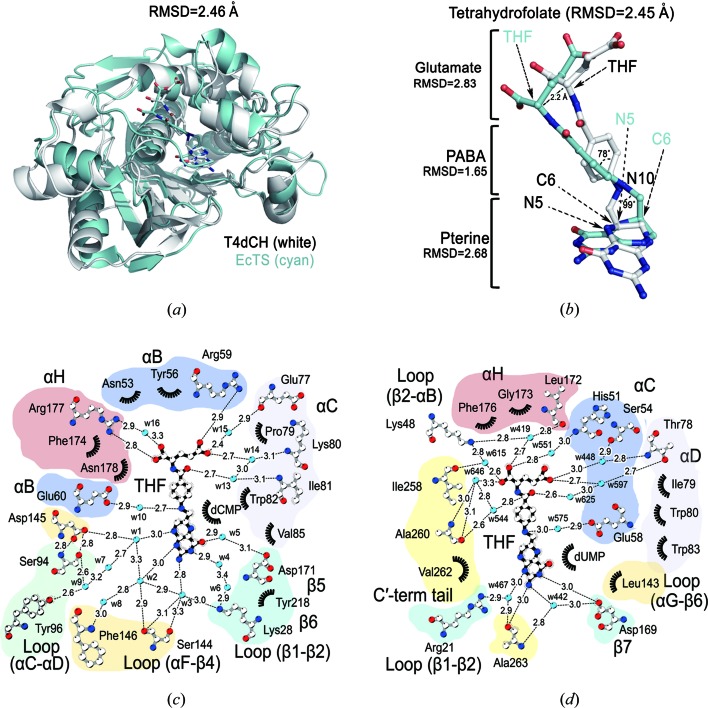
Structural comparison between the ternary complexes of T4dCH and EcTS. (*a*) Structural superposition of T4dCH (white) and EcTS (cyan). (*b*) Differences in the THF ligand geometries in the active sites of T4dCH and EcTS. This orientation was obtained from the optimal superposition of T4dCH and EcTS protein as shown in (*a*). Carbon atoms of THF bound to T4dCH and EcTS are colored white and cyan, respectively. (*c*) Schematic plot of the interaction between T4dCH and THF. (*d*) Schematic plot of the interaction between EcTS and THF. The dotted lines and numbers indicate the hydrogen bonds and their distances in Å, respectively. The starburst indicates the hydrophobic interactions. Water molecules are labeled as ‘w’ for clarity. Nitrogen and oxygen atoms are colored blue and red, respectively, for all panels.

This is the first structure of the enzyme–substrate–cofactor ternary complex in the class of pyrimidine hydroxymethylases, and the most characterized enzyme in the class of pyrimidine methylases is EcTS. Therefore, we compared the molecular geometry of THF in each of the ternary complexes. The calculated RMSD value is 2.46 Å for 204 matching Cα atoms between T4dCH and EcTS [Fig. 3 ▸(*a*)]. Although superposed structures show deviations in many parts, there is high overlay of the catalytic nucleophiles and pyrimidine monophosphate, the substrate. Interestingly, the sub-parts of the THF molecule also show distinct differences between the structures [Fig. 3 ▸(*b*)]. First, the pterin ring of THF in T4dCH rotates to the outside of the active site by 60° in the same plane with an RMSD of 2.68 Å. This rotation induces differences in the atomic positions of C6 and N5, which deviate by 1.7 and 2.0 Å, respectively. Interestingly, C6 is a critical site for acting as a hydrogen-bond donor in the last step of the TS reaction, so these differences may explain why dCH triggers hydroxylation and not a hydride transfer reaction. Pterin ring rotation also affects the atomic position differences of the 4-oxygen and 2-amino groups, which deviate by 4.0 and 4.4 Å, respectively. The PABA group shows a relatively low RMSD value of 1.65 Å, since the C1′—N10 bonds are aligned almost in the same position. The N10 atom in the PABA group is critical to mTHF activation via water-mediated protonation by glutamate residues (Glu60 in T4dCH and Glu58 in EcTS). dCH shares the same methyl­ation step, which uses mTHF protonation as the starting point of the reaction. Thus, the atomic position of N10 must be highly conserved between the ternary complexes. The N10—C9 bond is tilted by 99°, and the C9 position deviates by 2.4 Å and is linked to the rotated pterin core and the PABA ring. The PABA ring is also tilted by 78° away from the C1′—N10 axis, showing that dCH recognizes the PABA ring using different hydrophobic residues compared with EcTS. Finally, the glutamate tail shows the highest RMSD value of 2.83 Å; however, the side-chain direction does not differ between T4dCH and EcTS.

To analyze the differences occurring with the THF ligand geometry, we compared THF binding to T4dCH [Fig. 3 ▸(*c*)] with that of EcTS [Fig. 3 ▸(*d*)]. The THF binding site is composed of three α-helices (α-helix B, C, H in T4dCH and C, D, H in EcTS) that recognize the PABA ring and glutamate tail of THF. The residues recognizing THF are not matched between T4dCH and EcTS in their tertiary structures. For example, the main-chain of Tyr56 in α-helix B of T4dCH is aligned to that of Ser54 in α-helix B of EcTS. However, the aromatic side chain of Tyr56 contributes to PABA ring recognition via a hydrophobic interaction, and Ser54 interacts with the glutamate tail mediated by a water molecule. The side-chain position of Tyr56 in T4dCH overlays with Ile79 in α-helix C of EcTS, which forms a hydrophobic interaction with the PABA ring. Regarding hydrophilic interactions, the basic side chain of Arg177 in α-helix H of T4dCH overlays with Lys48 in EcTS; although, the main chain of Arg177 in T4dCH is superposed with that of Pro175 in the primary and tertiary structures of EcTS.

A major structural difference between T4dCH and EcTS is the lack of the C-terminal tail in T4dCH. In the ternary complex of EcTS, the C-terminal tail (Ile258, Ala260, Val262 and Ala263) plays a key role in recognizing all of the subparts of THF and contributes to sealing the active site for the methylation reaction by maintaining a hydrophobic environment. In the T4dCH structure, the extremely short C-terminal tail cannot contribute to the sealing of the active site; thus, we have identified the residues that recognize THF in T4dCH which correspond to the C-terminus of EcTS. In EcTS, Ile258 and Ala260 recognize the glutamate tail by water-mediated hydrogen bonds [Fig. 3 ▸(*d*)]. In T4dCH, the bifurcated side-chain guanidium group of Arg177 recognizes the glutamate tail directly as well as indirectly via a water-mediated interaction [Fig. 3 ▸(*c*)]. The main chain of Ala260 in EcTS contributes to recognizing the oxygen atom of PABA via a water-mediated interaction, and similarly, the main chain of Ile81 in α-helix C of T4dCH recognizes the oxygen atom via a water-mediated interaction. Unexpectedly, Ile81 also contributes to pterin recognition by hydrophobic interactions, including one with Val262, in EcTS. Lastly, the main chain of Ala263 in EcTS recognizes the pterin ring, and this interaction is substituted by polar interactions of the side-chain hydroxyl group of Ser144 and the main chain of Phe146 in T4dCH. In summary, despite the lack of a C-terminal tail, T4dCH also recognizes the same cofactor, THF, using different residues in the THF binding pocket. This feature would provide the plasticity for efficient rearrangement of the ligand conformation during hydroxy­methylation.

We also looked at differences in the orientation of the PABA ring, because gate opening of the PABA-ring binding site is critical for binding and releasing THF. There are hydrophobic interactions for accommodating the PABA ring. Tyr56, Ile81 and Phe174 in dCH, and Ile79, Gly173 and Phe176 in EcTS are critical to the hydrophobic interaction. These residues are also not matched between the tertiary structures of EcTS and T4dCH. For example, Ile81 does not exist in EcTS, and thus, it has a different PABA ring orientation in the active pocket to T4dCH [Fig. 3 ▸(*b*)]. Despite the difference in the PABA ring orientation, there is a highly conserved glutamate residue (Glu60 in T4dCH and Glu58 in EcTS) for recognizing the N10 position for water-mediated activation of mTHF. In the enzymatic process, the rotation of the pterin core is critical for the reaction steps occurring between hydroxylation and the hydride transfer. There is no direct interaction of C6 of the pterin ring, such as that with the hydride donor observed in EcTS. Thus, recognition of the amine groups of the pterin ring must be critical for its orientation. When comparing the pterin core interactions in the active sites of T4dCH and EcTS, we observed enrichment of ordered solvent molecules that form the water-mediated hydrogen-bonded networks using hydrophilic residues in the β-strands and loop regions of T4dCH. However, in EcTS, the water molecules are highly restricted in the active site and only three residues (Arg21, Asp169 and Ala263) recognize the amine groups of the pterin core. In particular, Asp169 interacts with N3 and the 2-amino group of the pterin ring, and is already known as a critical residue for cofactor binding and orientation of hydride transfer. The corresponding residue in T4dCH is Asp171, which instead recognizes a carbonyl oxygen of the pterin ring via a water-mediated hydrogen bond. Moreover, it is already known that Trp83 of EcTS controls the solvent accessibility of the active site, and Leu143 supports the side-chain orientation of Trp83 (Fritz *et al.*, 2002 ▸). Interestingly, the residues corresponding to Trp83 and Leu143 in EcTS are Val85 and Asp145 in T4dCH, which may play different roles. Val85 has a shorter hydrophobic side chain than the bulky tryptophan; thus, T4dCH requires additional hydrophobic interactions to compensate. This is supported by the finding that Ile81 in T4dCH is also involved in the recognition of the pterin ring. Moreover, Asp145 does not contribute to the hydrophobicity, but participates in an intramolecular, ionic interaction with the main chain of Ser94. It also provides a negative charge to the inner surface of the active site using oxygen moieties of the side chains. Furthermore, Asp145 recognizes the N8 position of the pterin ring via a water-mediated hydrogen bond [Fig. 3 ▸(*c*)]. This interaction might be critical for pterin rotation by preventing the hydride transfer reaction, and instead, attracting a water molecule to the C7 position of the exocyclic methylene intermediate state of dCMP. To test this hypothesis and to further enhance our understanding of the hydroxylation mechanism of dCH, we performed a mutagenesis study.

### 3.5. Mutational analysis of T4dCH   

To determine the mechanism of the hydroxymethylation reaction, we tested the activities of several T4dCH mutants that were designed based on our structures. The reaction product 5hm-dCMP can be easily monitored by HPLC at an absorption wavelength of 260 nm. There is a clear difference in retention times between the substrate, dCMP and the product, 5hm-dCMP. The identities of the eluents are then confirmed in two ways: by comparison with reference molecules and mass spectrometric analysis of the product. As the enzyme reaction proceeded, an increasing amount of reaction product (5hm-dCMP) and a decreasing amount of substrate (dCMP) were detected [Fig. S7(*a*)]. For the hydroxymethyl­ation reaction, a water molecule has to be activated and incorporated into the product during synthesis. As shown in the structure [Fig. 2 ▸(*c*)], the potential candidates for these water molecules are Wat1, Wat7 and Wat9, because these ordered solvents are located between the catalytic nucleophile and estimated C7 position of the exocyclic methylene intermediate of dCMP. The polar residues – Ser94, Tyr96 and Asp145 – that interact with these waters could be critical for hydroxylation. Ser94 in T4dCH is equivalent to Val93 in EcTS in the tertiary structures. Thus, we generated two mutants: S94A to eliminate the polar group that interacts with the water and S94N to introduce a longer side chain to clash with a water molecule. Tyr96 in dCH is equivalent to Tyr94 in EcTS, which is an essential base for removing C5-H from intermediates. However, the positions of the hydroxyl group of Tyr96 in T4dCH and of Tyr94 in EcTS deviate by approximately 2.0 Å. The consequence of this has not yet been investigated biochemically in the pyrimidine hydroxymethylase class. Thus, we mutated this tyrosine to phenylalanine. Asp145 is a unique polar residue in the active site of T4dCH, and we mutated it to asparagine to understand the effects of the negatively charged side chain. As negative controls, two mutants, C148S and D179N, were also generated. Cys148 and Asp179 are known nucleophiles required for generating the dCMP-conjugated adduct and acting as specificity determinants for the cytidine ring and catalysis, respectively (Graves *et al.*, 1992 ▸). As expected, the C148S and D179N mutants produced negligible amounts of 5hm-dCMP [Figs. S7(*b*) and S7(*c*)], and all of the other mutants (S94A, S94N, Y96F and D145N) showed significant deficiences in enzyme activity (Fig. S7). Interestingly, S94A possessed residual activity of approximately 25% [Figs. S7(*d*) and S7(*h*)], while the mutation introducing the longer side chain with the same polar property (S94N) completely blocked any activity [Fig. S7(*e*)]. These results suggest that Ser94 coordinates a critical water for the enzyme reaction. Wat9 is located at the position of the hydroxyl group of Tyr94 in EcTS, whereas in T4dCH, it exists between Ser94 and Tyr96, which bind the water tightly on each side. Similarly, the Y96F mutant did not produce any product [Fig. S7(*f*)]. Asp145 plays dual roles within the structure: the first is maintaining the position of Ser94 through an ionic interaction and the second is controlling the orientation of the pterin core through a water-mediated (Wat1) interaction. Moreover, this water might be a catalytic substrate for hydroxylation. Replacement of the acidic carboxylate with the polar carboxamide group completely abolished the catalytic activity of T4dCH [Fig. S7(*g*)]. We conclude that the carboxylate of Asp145 plays a critical role in activating the water (Wat1), and then the activated hydroxide is inserted into the methylene intermediate of 5m-dCMP.

### 3.6. Binding of dCMP to T4dCH mutants   

Although the activity assay results described in the previous section provide insights into the role of particular residues, next we wanted to determine whether the lack of reaction product originated from a catalytic defect caused by mutations of the residues or a lack of substrate binding. To address this question, we measured the binding constants of WT and mutant T4dCH constructs by ITC. The dissociation constant (*K*_D_) for WT binding to dCMP was 10.2 µ*M* [Fig. S8(*a*)], with favorable enthalpic and entropic parameters [Fig. S9(*a*)]. The *K*_D_ values for dCMP binding to all of the mutants were measured (Fig. S8 and Table 2 ▸). The S94A and D145N mutants showed no detectable binding affinity. However, mutation of the catalytic residue C148S did not have an appreciable effect on binding affinity compared with WT. The mutation of the pyrimidine base determinant D179N caused an approximately 13-fold reduction in binding affinity. Compared with the energetic parameters of WT, the well known mutants C148S and D179N have similar entropic changes, but reduced enthalpic changes upon dCMP binding [Figs. S9(*b*) and S9(*c*)]. The lowered binding affinity (with only slight lowering in the case of C148S) is caused by the reduction in enthalpy-driven forces. Interestingly, the *K*_D_ values of S94N and Y96F mutants were reduced by approximately eightfold and twofold, respectively (Table 2 ▸). These mutations caused unfavorable entropy changes upon dCMP binding (Fig. S9).

In addition to the binding affinity assay by ITC, we performed a thermal-shift assay using T4dCH WT and mutant constructs incubated with increasing amounts of dCMP (Fig. S10). Upon dCMP binding, the *T*_m_ values were elevated. However, S94A and D145N showed very minor differences in their thermal-shift profiles in response to dCMP [Figs. S10(*b*), S10(*e*) and S10(*h*)], and the extents of the thermal shifts were varied among mutants. All thermal-shift data are consistent with the ITC results (Fig. S8). From these two sets of data, we were able to interpret the activity results of the mutants in a more rational way. The C148S mutant is defective in generating the dCMP–enzyme adduct, which is critical for the enzyme reaction, although there is little change in the binding affinity. The D179N mutation significantly decreased the binding affinity to dCMP because the carboxylate side chain is a key determinant for the cytosine base. Moreover, it affects the electronic nature of the pyrimidine ring, which appears to be important for stacking with the pterin group of the mTHF cofactor [Fig. 2 ▸(*b*)]. Ser94, Tyr96 and Asp145 are located on the opposite side of Asp179 and near the hydroxy­methylation site. Intriguingly, mutation of Ser94 and Tyr96 causes entropic defects in dCMP binding (Fig. S9). The hydroxyl group of Tyr96 forms a hydrogen bond with the amine group attached to the C4 atom of the pyrimidine ring; this is dissimilar to TS, because the oxygen atom at C4 in the uracil ring cannot be recognized by the hydroxyl oxygen of tyrosine. The main-chain nitrogen and side-chain hydroxyl oxygen of Ser94 form a polar interaction with the side-chain carboxylate of Asp145. Furthermore, a potential key water molecule (Wat1) is located within hydrogen-bonding distance of the carboxylate of Asp145 as well as the N1 atom of the pterin ring [Fig. 3 ▸(*c*)]. The geometry of the dCMP substrate, mTHF cofactor, Wat1, Ser94 and Asp145 appears to be critical for the hydroxy­methylation reaction. Furthermore, the Ser94 and Asp145 residues located in the flexible loop region appear to be essential for maintaining the binding of dCMP and hydroxy­methylation.

## 4. Discussion   

Our high-resolution substrate–cofactor ternary complex structure of T4dCH in combination with mutational and biochemical data has provided an understanding of the mechanism of pyrimidine hydroxymethylation and insights into identifying dCH proteins from various species within the CH and TS groups. The former includes how dCH recognizes THF – despite the lack of the C-terminal tail that is characteristic of TS – and which water molecules are critical for hyroxymethylation. Thus, substrate specificities among TS family members can be identified by comparing key determinant residues within a sequence alignment.

**Figure 4:**
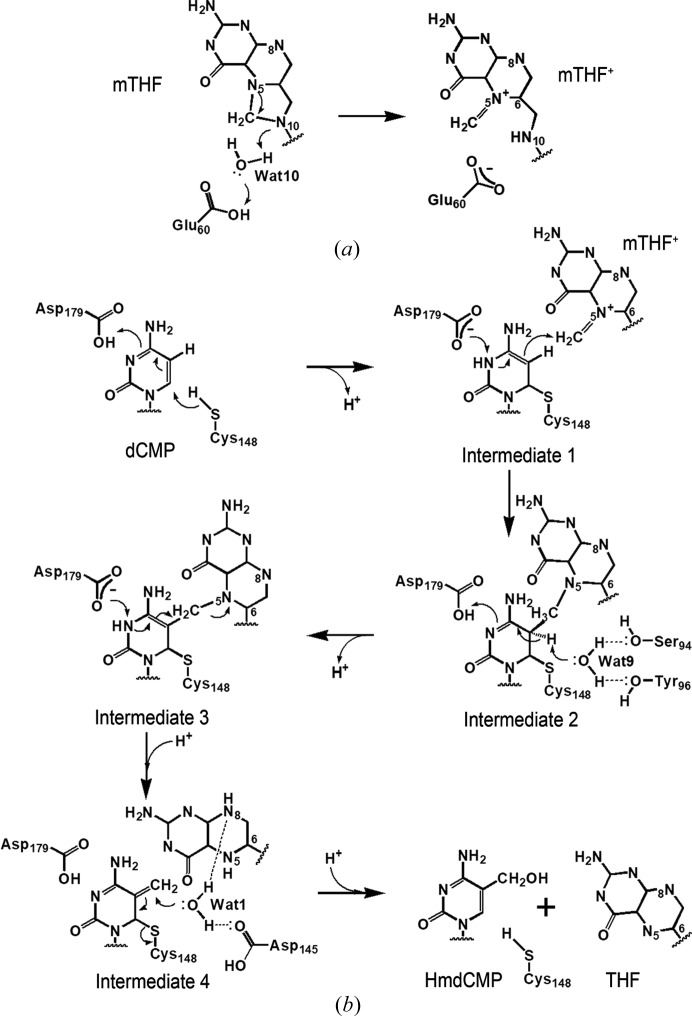
Reaction mechanism of T4dCH. (*a*) Schematic diagram showing the proposed mechanism of cofactor activation. For methyl-group transfer, the mTHF is converted to protonated mTHF^+^ by a water (Wat10) that had been activated by Glu60. The electron flows are indicated by arrows. (*b*) Schematic diagram showing the proposed mechanism of hydroxy­methylation by T4dCH. The intermediates 1, 2, 3 and 4 are shown. The hydrogen-bonding interactions are represented as dotted lines.

A previously proposed enzyme mechanism was based on the structural similarity of the active sites between dCH and TS (Butler *et al.*, 1994 ▸) since TS is one of the most well characterized enzymes (Finer-Moore *et al.*, 2003 ▸). As described, T4dCH possesses many unique features for accommodating THF cofactors and ordered waters, which appear to be critical for the enzyme reaction. Now, we propose an updated mechanism of dCMP hydroxymethylation based on our ternary complex structure of T4dCH and previous studies of TS (Hardy *et al.*, 1995 ▸; Graves *et al.*, 1992 ▸; Butler *et al.*, 1994 ▸; Graves & Hardy, 1994 ▸; Carreras & Santi, 1995 ▸; Kamb *et al.*, 1992 ▸; Fritz *et al.*, 2002 ▸; Finer-Moore *et al.*, 2003 ▸; Perry *et al.*, 1993 ▸; Agarwalla *et al.*, 1997 ▸). For a single methyl group donation, mTHF has to be converted into mTHF^+^; this step is conserved between dCH and TS [Fig. 4 ▸(*a*)]. Glu60 (Glu58 in EcTS) activates a water molecule (Wat10 in our structure) and triggers a ring opening to produce mTHF^+^, which is used for the subsequent reaction. The pre-reaction step of substrate– cofactor binding is critical for recognition of the cytosine base by Asp179, and the phosphate group of dCMP by Arg123 and Arg124 from the opposite protomer (Song *et al.*, 1999 ▸). Then, the cofactor mTHF^+^ can bind to the active site of T4dCH when the hydrophobic PABA-binding site becomes opened by side-chain movement (Fig. S5). Finally, the subsequent hydroxymethylation steps are as shown in Fig. 4 ▸(*b*).

The first reaction step is nucleophilic attack of C6 of dCMP by Cys148. For the formation of intermediate 1, Asp179 participates in not only the recognition and orientation of cytosine, but also protonation of the N3 of the cytosine ring. This protonation step must be critical for the subsequent enzyme reaction and the stacking interaction between dCMP and mTHF^+^. Next is the formation of the T4dCH–dCMP–mTHF adduct (intermediate 2), which is a covalent conjugate of the protonated mTHF at the C5 position of intermediate 1. A water (Wat9) is coordinated by the hydroxyl moieties of Tyr96 and Ser94, and the main-chain nitrogen of Cys148, which indicates that the position of this water is tightly controlled by the enzyme itself. It abstracts a proton from C5 of dCMP generating intermediate 3. Next, the exocyclic methylene intermediate of dCMP–dCH (intermediate 4) is formed by THF elimination of intermediate 3. In the final step, a substrate water (Wat1) activated by Asp145 and N8 of the pterin ring is incorporated into the C7 exocyclic methylene position. Note, that in this reaction step, the hydride transfer, which occurs in TS, is impossible because the distance from the putative C7 exocyclic methylene position (hydrogen acceptor) to C6 of THF (hydrogen donor) is too far (4.7 Å apart) and hydrogen tunneling is blocked by the water molecule (Wat1). In our ternary complex structure, the distance from C6 of dCMP to the putative N5 methyl position of the pterin ring is also too far for direct methyl transfer (this cannot be measured exactly due to the lack of structural information on the mTHF complex), and thus, geometrical changes in THF must occur during the reaction steps. Finally, the cofactor THF and the product 5hm-dCMP are released from the active site. Incorporation of the hydroxymethyl group into dCMP might alter the water-mediated hydrogen-bonded networks and the chemical environment in the active site for easier dissociation of the ligands.

The TS enzyme utilizing dUMP as a substrate is an essential universal enzyme in living organisms; however, dCH is usually found in bacterial viruses. Therefore, TS has been studied extensively, and most of the pyrimidine modifying enzymes have been classified in the TS superfamily. However, many of the gene products found in bacterial viruses cannot be easily distinguished as a dCH from the TS superfamily (including TS and CH), an example of which is MilA, which possesses substrate specificity toward CMP (Zhao *et al.*, 2016 ▸). One of the fundamental differences between TS and dCH (or CH) is the formation of different products by hydroxymethylation using the same cofactor, mTHF–DHF for TS and THF for dCH (or CH). Therefore, for efficient turnover of the enzyme, some of the TS must exist as a fusion protein with DHFR, which is frequently found as bifunctional DHFR–TS in protozoans (Ivanetich & Santi, 1990 ▸; Carreras & Santi, 1995 ▸). We observed a hydrophobic gate for accommodating THF, which might be a special feature for regulating the entrance and release of the cofactor without using a C-terminal tail (Fig. S5). The residues involved (Ile81, Val85 and Phe174) in T4dCH are relatively conserved among the putative dCH species, and their hydrophobic nature is somewhat maintained in CH and TS (Fig. S11). Therefore, it is not a clear indicator for discriminating dCH proteins from the TS family. Previous studies clearly showed that Asp179 (in T4dCH) is a key determinant for substrate specificity of dCMP against dUMP (Graves *et al.*, 1992 ▸; Agarwalla *et al.*, 1997 ▸; Song *et al.*, 1999 ▸). The binding affinity of the D179N mutant for dCMP was approximately 13-fold lower based on our ITC experiments (Table 2 ▸). The specificity of CH to CMP against dCMP was also clearly shown by the structural and biochemical study of MilA from *Streptomyces rimofaciens* ZJU5119 (Li *et al.*, 2008 ▸; Zhao *et al.*, 2016 ▸), wherein double mutation of A176S and K133R altered the substrate preference of MilA from CMP to dCMP.

This ternary complex structure enabled us to identify key determinant residues for the hydroxymethylation reaction of dCH. Thus, we can easily distinguish hydroxymethylases from other members of the TS family. We aligned the key signature sequence regions of potential dCH family with TS (EcTS and T4TS) and CH (ScMilA) (Fig. S11). There is a highly conserved aspartic acid (Asp145 in dCH) that is three residues upstream of the catalytic cysteine (Cys148 in dCH). Asp145 in some phages is replaced with a functionally conserved glutamic acid which might play the same role. However, our mutational study showed that even a conservative mutation such as D145N cannot produce 5hm-dCMP [Fig. S7(*g*)]. The corresponding residue in TS enzymes is a hydrophobic leucine residue (Fig. S11). Asp145 plays a critical role in activating a substrate water that enables hydroxymethylation to occur, and furthermore, it interacts with the Ser94 residue, which is also highly conserved within the dCH family. The serine residue is replaced with the threonine residue in some bacteriophage species (Fig. S11), and the hydroxyl moiety of the threonine might interact with the aspartate or glutamate, as shown in the Ser94–Asp145 interaction found in T4dCH [Fig. 3 ▸(*c*)]. For the formation of intermediate 3, the side-chain hydroxyl moieties of Ser94 and Tyr96 are critical for water-mediated proton abstraction [Fig. 4 ▸(*b*)]. Therefore, the aspartate residue for activating a substrate water (three residues upstream of the catalytic cysteine) and the special geometries of the interacting serine and tyrosine residues (two residues after the serine) are thought to be a signature for hydroxy­methylation. To completely understand the hydroxymethyl­ation reaction by dCH, high-resolution structures of various mutants are necessary, as shown herein with TS. Nevertheless, the current T4dCH structure is the first example of a substrate–cofactor–enzyme ternary complex for pyrimidine hydroxymethylase. Thus, it provides the fundamental basis for performing functional studies to understand the pyrimidine modification system. 

## Supplementary Material

Supporting figures (S1-S11). DOI: 10.1107/S2052252518018274/lz5023sup1.pdf

PDB reference: apo T4dCH (I-SAD phasing), 6a9b

PDB reference: ternary complex (T4dCH–dCMP–THF), 6a9a

## Figures and Tables

**Table 1:** Data collection and refinement statistics FOM = figure of merit; DM = density modification. Values in parentheses are for the highest-resolution shell.

	apo WT (I-SAD)	C148S/D179N–dCMP–THF
PDB entry	6a9b	6a9a
		
Data collection		
X-ray source	Cu *Kα*	5 C, PAL
Wavelength (Å)	1.5418	0.9793
Space group	*I*222	*I*222
Cell dimensions		
*a*, *b*, *c* (Å)	52.60, 74.98, 155.34	52.63, 75.23, 154.09
α, β, γ (°)	90, 90, 90	90, 90, 90
Resolution (Å)	50-2.01 (2.04–2.01)	50-1.90 (1.93–1.90)
*R*_merge_	0.059 (0.208)	0.115 (0.627)
*R*_meas_	0.064 (0.225)	0.125 (0.679)
*R*_pim_	0.024 (0.085)	0.047 (0.248)
〈*I/σ*(*I*)〉	59.22 (11.9)	26.9 (3.3)
CC_1/2_	0.999 (0.979)	0.993 (0.856)
Completeness (%)	99.8 (100)	99.8 (100)
Redundancy	7.0 (6.8)	7.1 (7.3)
		
SAD phasing		
No. of I atoms	1	
Initial FOM	0.39	
FOM after DM	0.69	
		
Refinement		
Resolution (Å)	26.3–2.01	37.6–1.90
No. reflections	20 770	24 595
*R*_work_/*R*_free_ (%)	16.01/19.15 (18.18/22.34)	15.65/19.55 (20.40/26.27)
No. of atoms		
Protein	2009	2009
Ligands	6	53
Water	205	255
*B* factors (Å^2^)		
Protein	31.8	23.9
Ligands	32.9	41.4
Water	38.8	35.8
RMSD		
Bond lengths (Å)	0.007	0.009
Bond angles (°)	0.786	0.941
Ramachandran plot		
Favored (%)	96.31	97.13
Allowed (%)	3.69	2.87
Outliers (%)	0	0
Clashscore	3.53	2.88
*MolProbity* score	1.39	1.23

**Table 2:** Summary of the binding affinities of T4dCH to dCMP using ITC

Name	*N* (sites)	*K*_D_ (µ*M*)	Δ*H* (kcal mol^−1^)	Δ*G* (kcal mol^−1^)	−*T*Δ*S* (kcal mol^−1^)	[Syringe] (m*M*)	[Cell] (m*M*)	Temperature (°C)
WT	0.769	10.2	−2.86	−6.81	−3.96	1	100	25
S94A	N/A	N/A	N/A	N/A	N/A	1	80	25
S94N	0.454	80.4	−24.7	−5.59	19.1	1	100	25
Y96F	0.213	19.6	−47.5	−6.42	41.1	1	70	25
D145N	N/A	N/A	N/A	N/A	N/A	1	86	25
C148S	0.505	13.4	−2.24	−6.65	−4.41	1	100	25
D179N	0.364	129	−0.961	−5.31	−4.35	5	500	25
