# Supplementary material for: A cytosine modification mechanism revealed by the structure of a ternary complex of deoxycytidylate hydroxymethylase from bacteriophage T4 with its cofactor and substrate
Source: IUCrJ. 2019 Jan 24;6(Pt 2):206–17. doi: 10.1107/S2052252518018274 (PMC6400193; doi:10.1107/S2052252518018274)
Supplement: Supplementary file 1 [file m-06-00206-sup1.pdf]

IUCrJ

Volume 6 (2019)

Supporting information for article:

A cytosine modification mechanism revealed by the ternary complex structure of deoxycytidylate hydroxymethylase from bacteriophage T4 with its cofactor and substrate

Si Hoon Park, Se Won Suh and Hyun Hyu Song

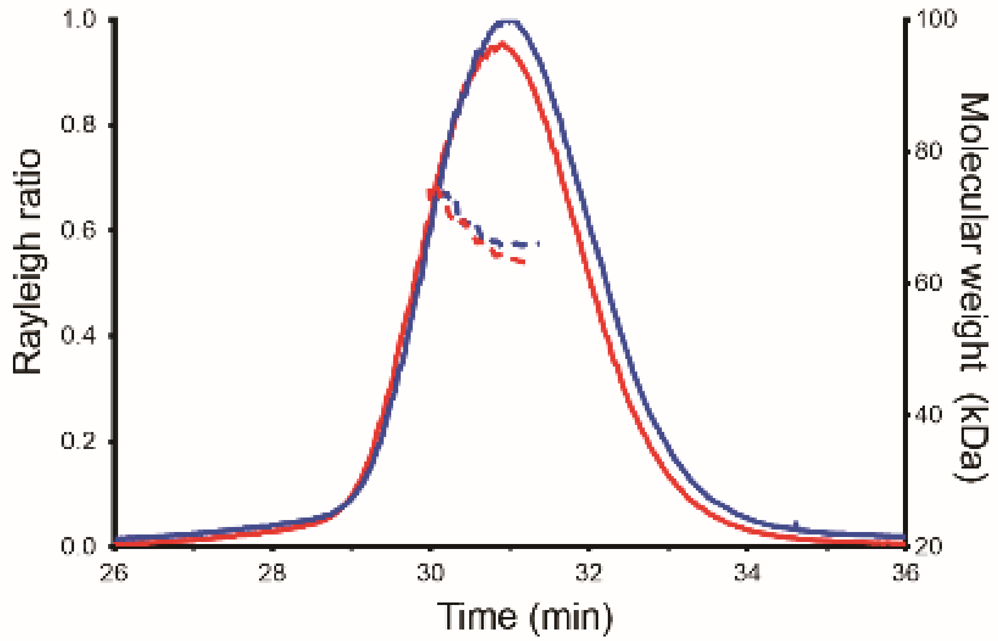

Name	M.W. (Experimental)	M.W. (Theoretical)
dCH-dCMP-THF	66.3 kDa	60.6 kDa
dCH-dCMP	64.7 kDa	59.7 kDa

Figure S1 Molecular weight change upon cofactor binding to T4dCH. Molecular weights of dimeric T4dCH in the presence (blue) or absence (red) of THF were analysed by SEC-MALS. Solid line represents the Rayleigh ratio and dotted line indicates average molecular weights. The table below is the summary of these SEC-MALS results.

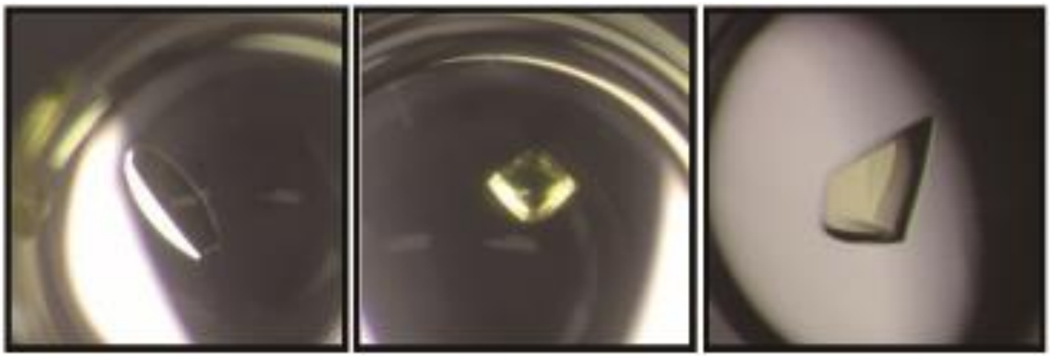

(*a*) (*b*) (*c*)

Figure S2 Color change of T4dCH crystals by soaking or co-crystallizing them with THF. (*a*) A transparent crystal of WT with dCMP. (*b*) A yellow-colored crystal of T4dCH WT with dCMP and THF. (*c*) A yellow colored crystal of the C148S/D179N mutant with dCMP and THF.

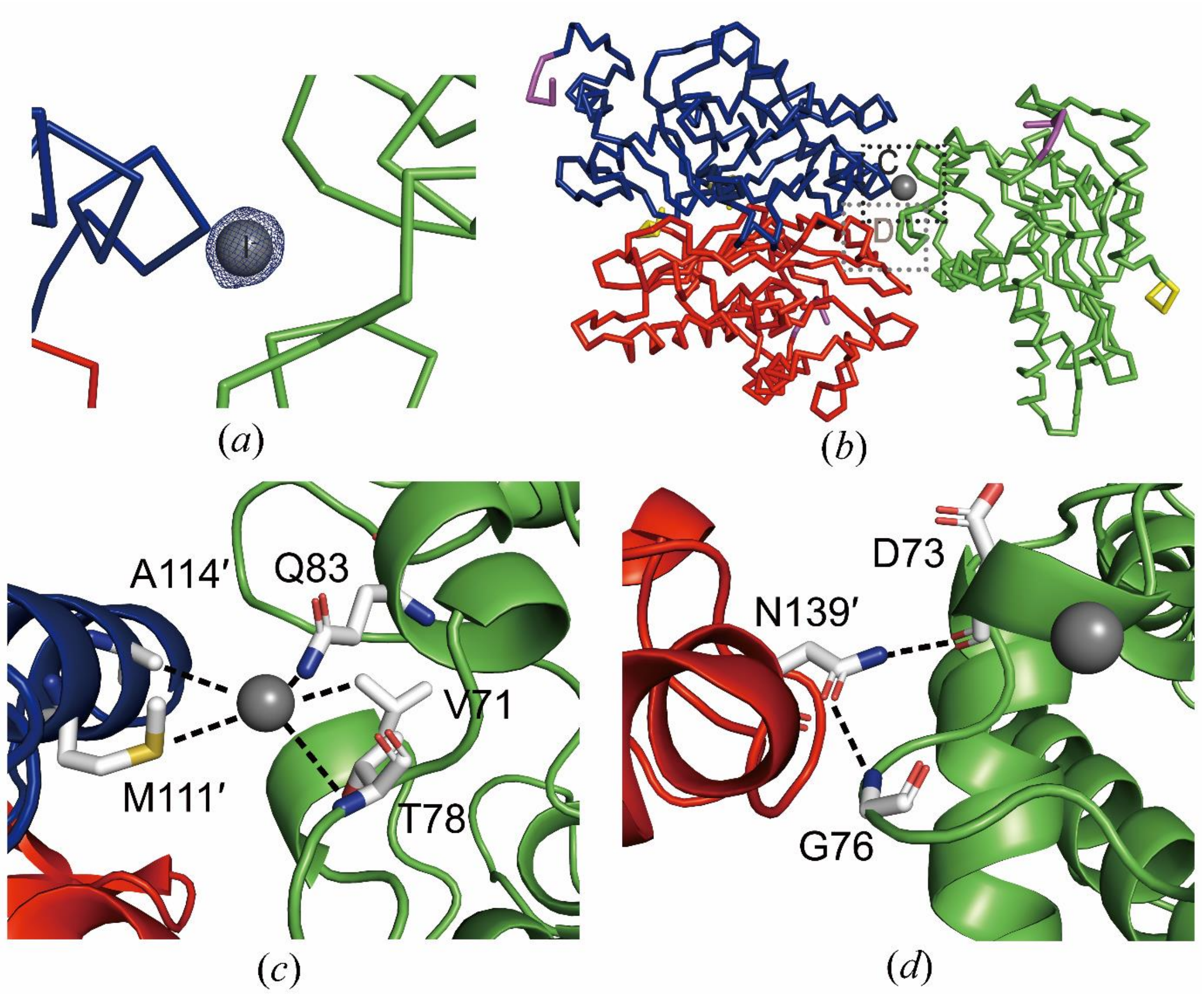

Figure S3 Iodide is critical for crystallizing the tetragonal form of T4dCH. (*a*) Anomalous difference Patterson map for the iodide involved in crystal packing. The iodide is shown as a grey ball and the blue mesh is contoured at 5.0σ. Each polypeptide chain of T4dCH near the iodide is represented as a stick model colored blue and green, respectively. (*b*) Crystal packing of the tetragonal form by iodide ion. The Cα stick models are shown in blue, red, and green for each protomer, respectively. N- and C-terminal regions are colored yellow and magenta, respectively. Black and grey dotted boxes indicate the regions shown in panels (*c*) and (*d*), respectively. (*c*) The residues directly interacting with iodide for tetragonal crystal packing. (*d*) The hydrogen bonding interaction induced by the bound iodide additionally stabilizes the crystal packing.

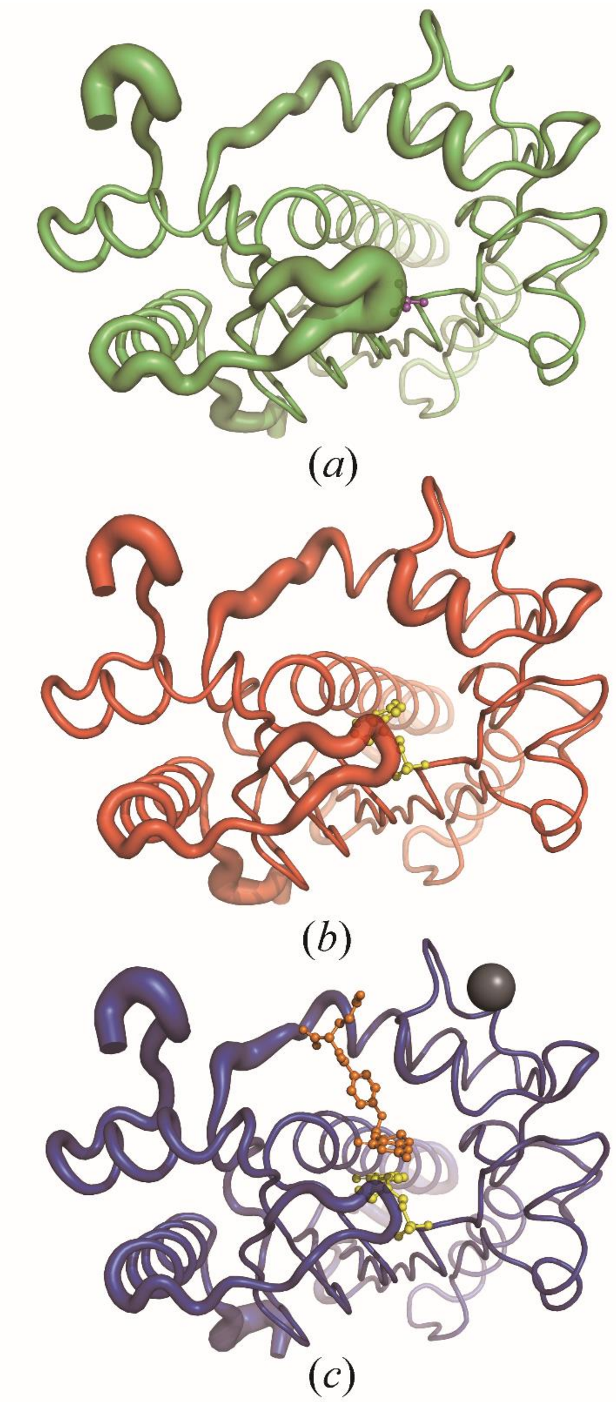

Figure S4 Reduced B-factors of T4dCH upon substrate and cofactor binding. (*a*) B-factor distribution of the *apo* (PO_4 bound) structure. (*b*) B-factor distribution of the binary complex structure of T4dCH and dCMP. (*c*) B-factor distribution of ternary complex structure in presence of iodide. In particular, the loop near the active site and an α-helix near the iodide show dramatic reductions in B-factor values in the presence of substrate and cofactor, which is represented by a narrow tube radius.

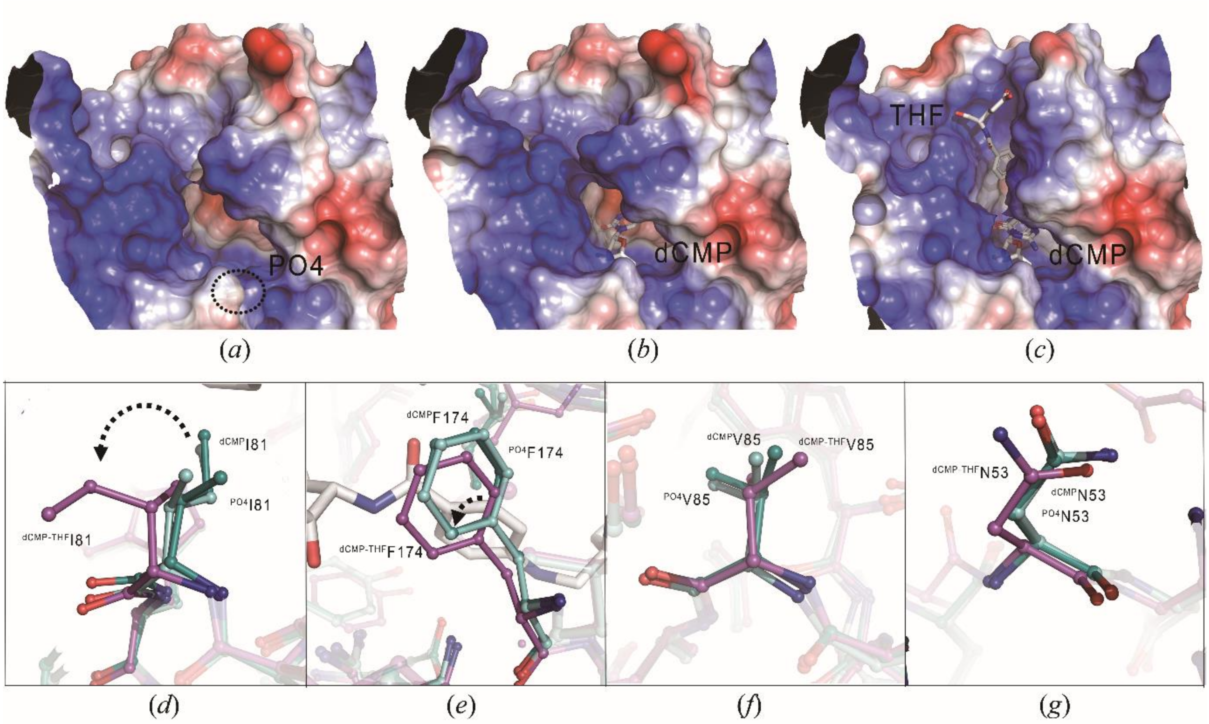

Figure S5 Local conformational changes of T4dCH upon complex formation. Electrostatic potential surface around the active site with ligands shown as sticks in the (*a*) apo form, (*b*) binary complex, and (*c*) ternary complex. Positively- and negatively-charged surfaces are colored blue and red, respectively. (*d*-*g*) Representative residues for triggering conformational changes of dCH. The carbon atoms in the ball and stick models of the apo form (PO4), binary complex (dCMP), and ternary complex (dCMP-THF) are colored cyan, teal green and magenta, respectively. Nitrogen and oxygen atoms are colored blue and red for all models.

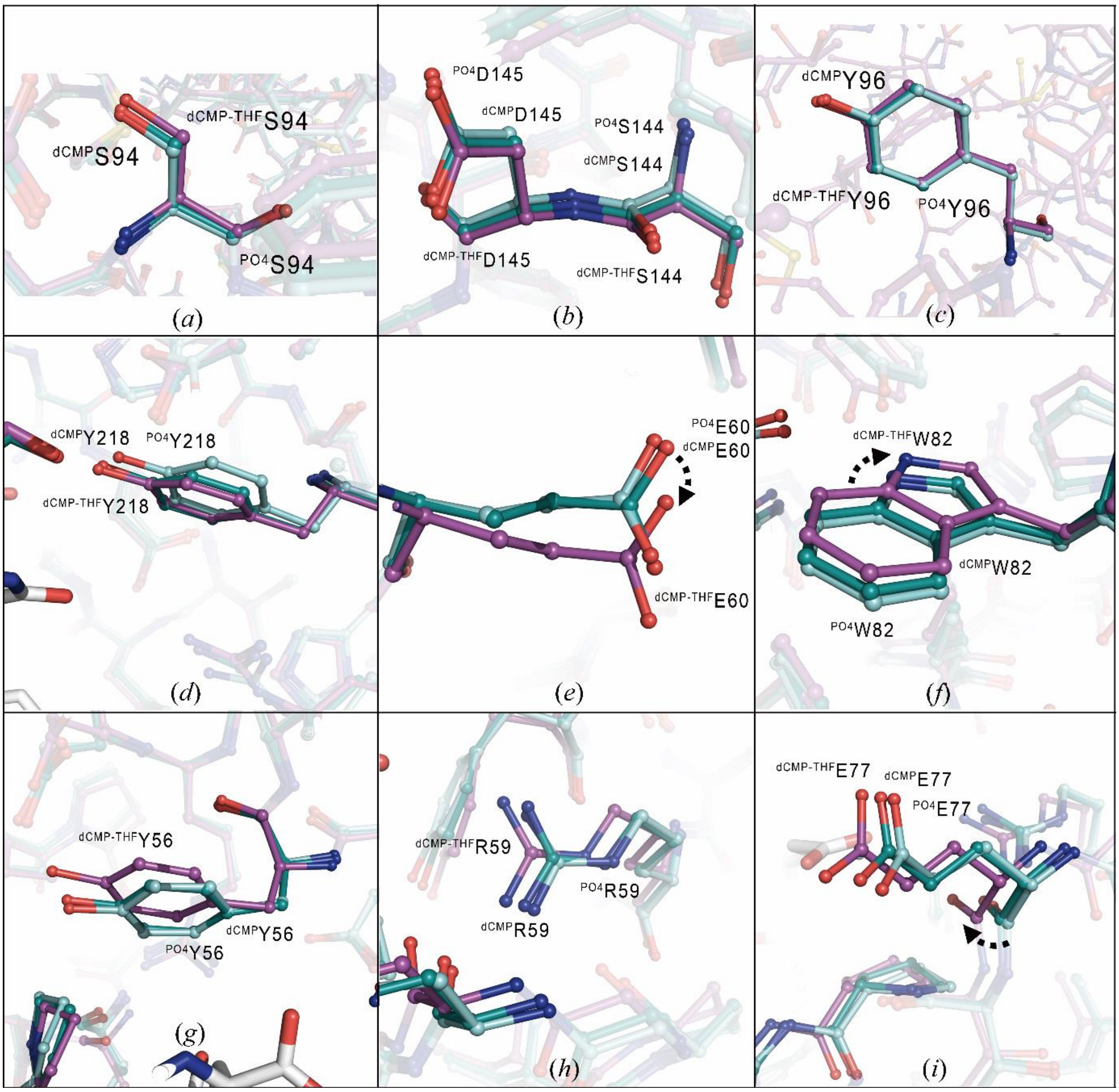

Figure S6 Local conformational changes of T4dCH upon complex formation. The carbon atoms in the ball and stick models of the *apo* form (PO_4), binary complex (dCMP), and ternary complex (dCMP-THF) are colored cyan, teal green, and magenta, respectively. Nitrogen and oxygen atoms are colored blue and red for all models. (*a*-*d*) Residues involved in the pterin recognition. (*e*-*g*) The side-chain movements of the PABA ring and the N10-recognition site of T4dCH. (*h* and *i*) Residual movements induced by glutamate tail binding.

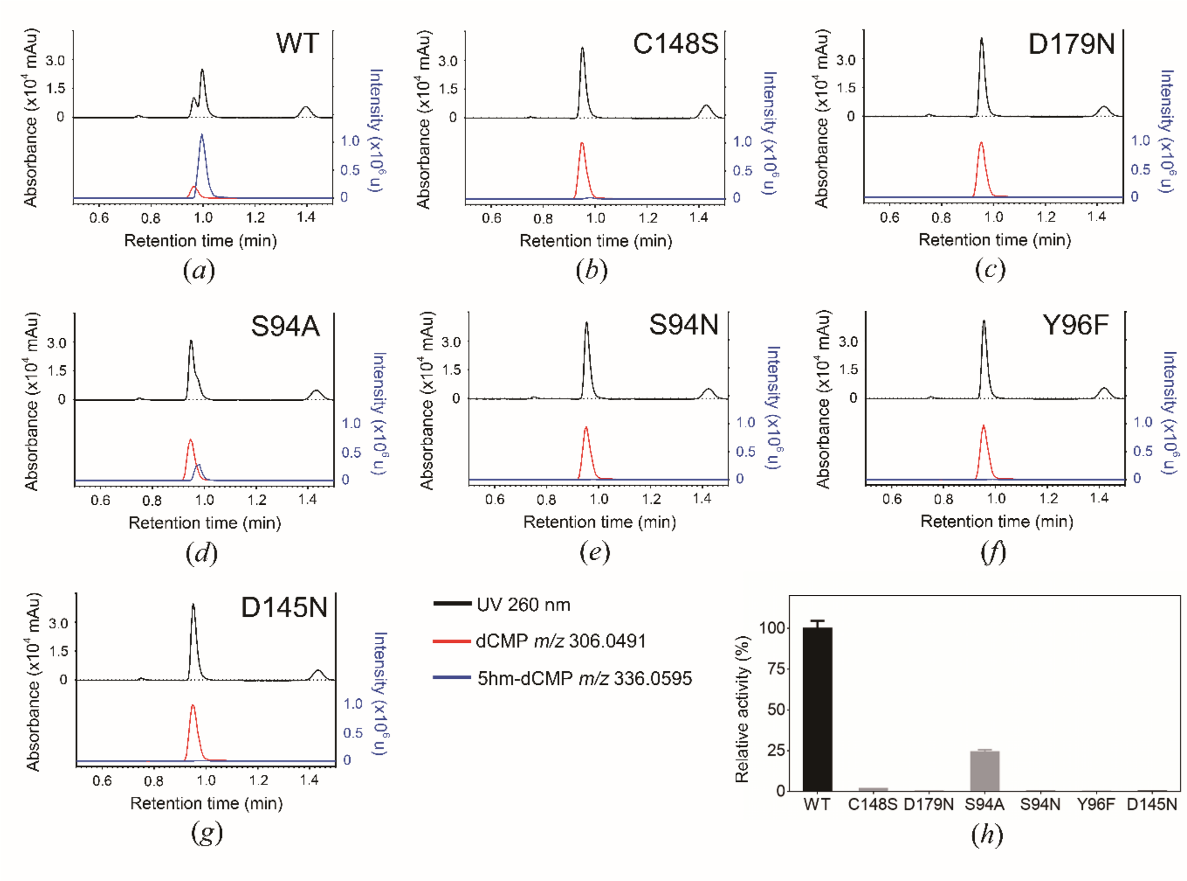

Figure S7 Enzymatic activities of WT and mutant T4dCH constructs. Measurement of the activities of T4dCH (*a*) WT, (*b*) C148S, (*c*) D179N, (*d*) S94A, (*e*) S94N, (*f*) Y96F, and (*g*) D145N. UV (260 nm) absorption spectra are shown as black lines (left axis). Mass spectra of dCMP (m/z 306.0491) and 5hm-dCMP (m/z 336.0595) are shown as red and blue lines, respectively. (*h*) The relative activities of WT (as 100%) and its mutants are shown as a bar graph. All experiments were performed in triplicate.

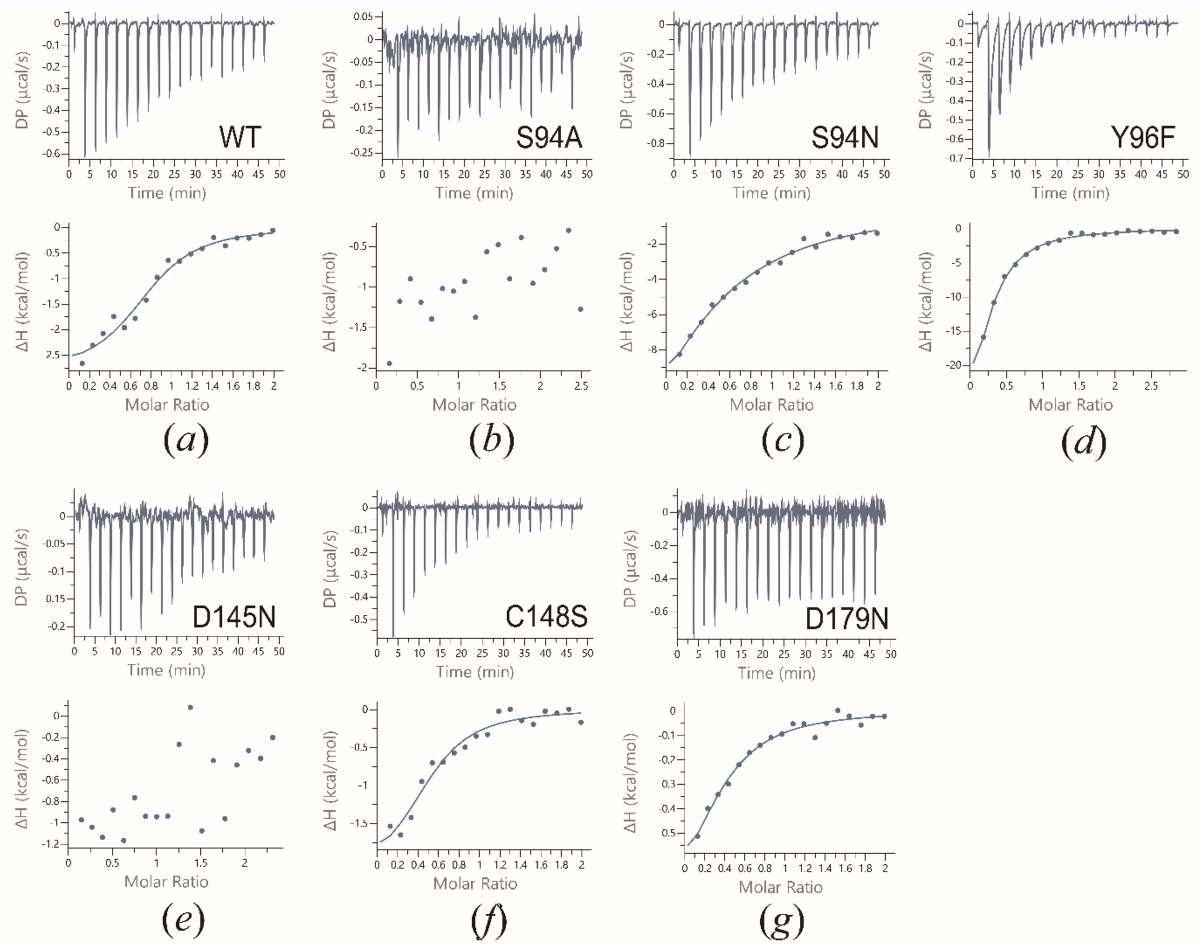

Figure S8 Binding affinities of T4dCH to dCMP. Measurement of the affinities of T4dCH (*a*) WT, (*b*) S94A, (*c*) S94N, (*d*) Y96F, (*e*) D145N, (*f*) C148S, and (*g*) D179N using ITC. With the exception of S94N and D145N, the thermodynamic parameters are shown in Supplementary Fig. S9.

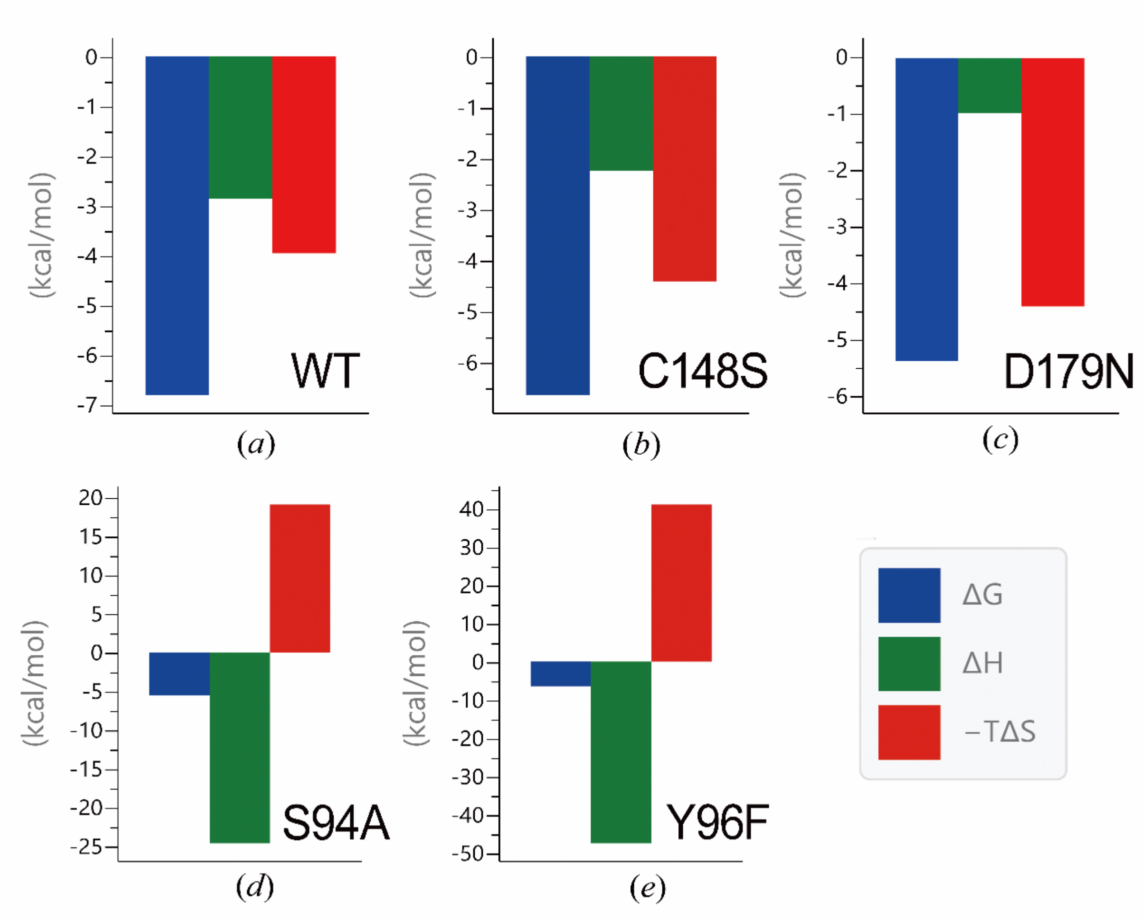

Figure S9 Thermodynamic parameters of T4dCH WT and mutant constructs upon binding of dCMP measured by ITC. The signature plots of (*a*) WT, (*b*) C148S, (*c*) D179N, (*d*) S94N, and (*e*) Y96F. The bars representing ΔG, ΔH, and -TΔS are colored blue, green and red, respectively.

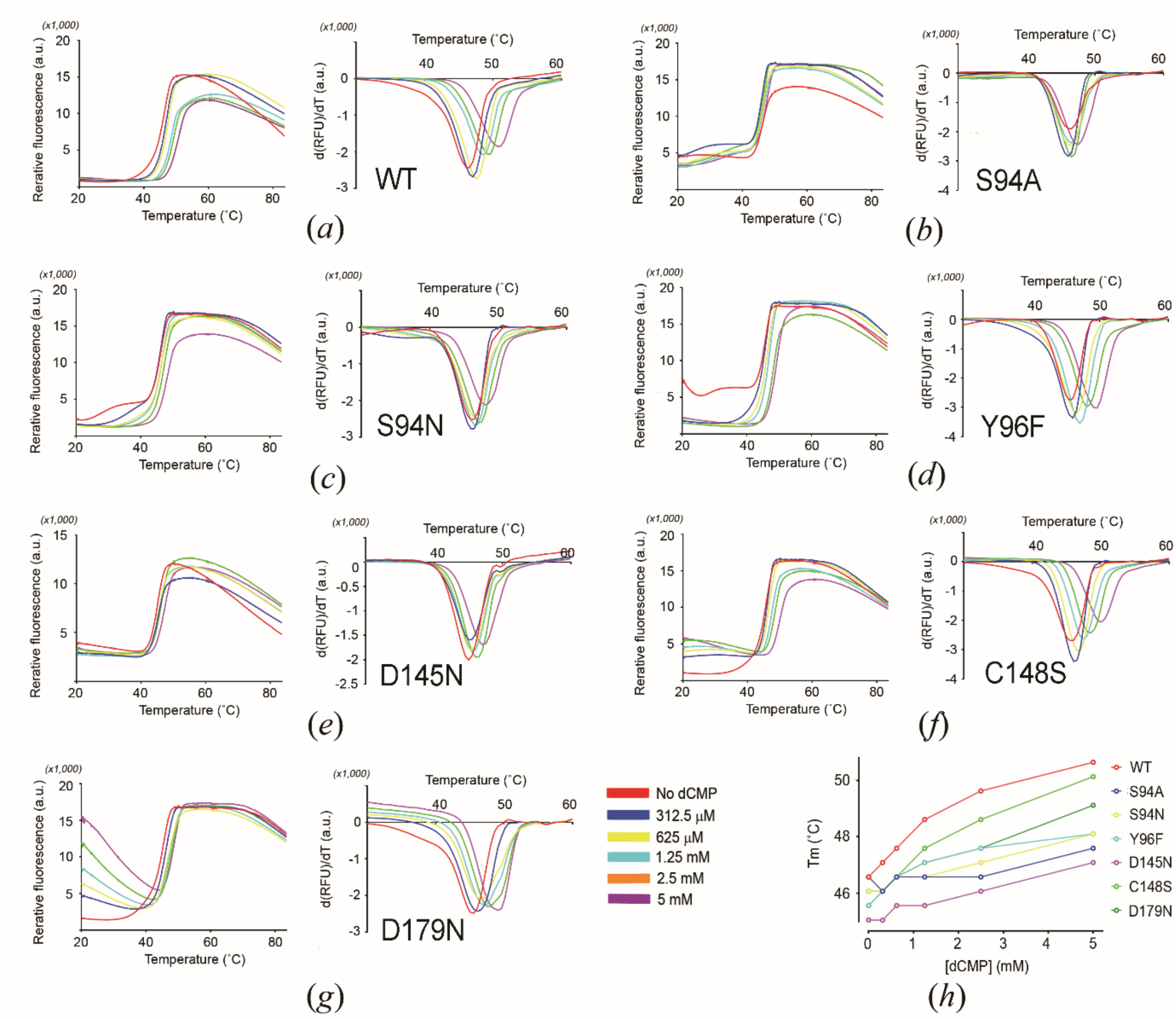

Figure S10 Thermal shift assay of T4dCH WT and mutant constructs showing enhanced T_m values with increasing dCMP concentrations. Melting curves and peak diagrams of (*a*) WT, (*b*) S94A, (*c*) S94N, (*d*) Y96F, (*e*) D145N, (*f*) C148S, and (*g*) D179N were measured with various concentrations of dCMP. Red, blue, yellow, cyan, orange, and purple lines represent constructs incubated with 0, 0.3125, 0.625, 1.25, 2.5, and 5 mM dCMP, respectively. (*h*) Summary of T_m changes of WT and mutant constructs incubated with increasing amount of dCMP. Red, blue, yellow, cyan, purple, light green, and green lines represent the data for WT, S94A, S94N, Y96F, D145N, C148S, and D179N mutants, respectively.

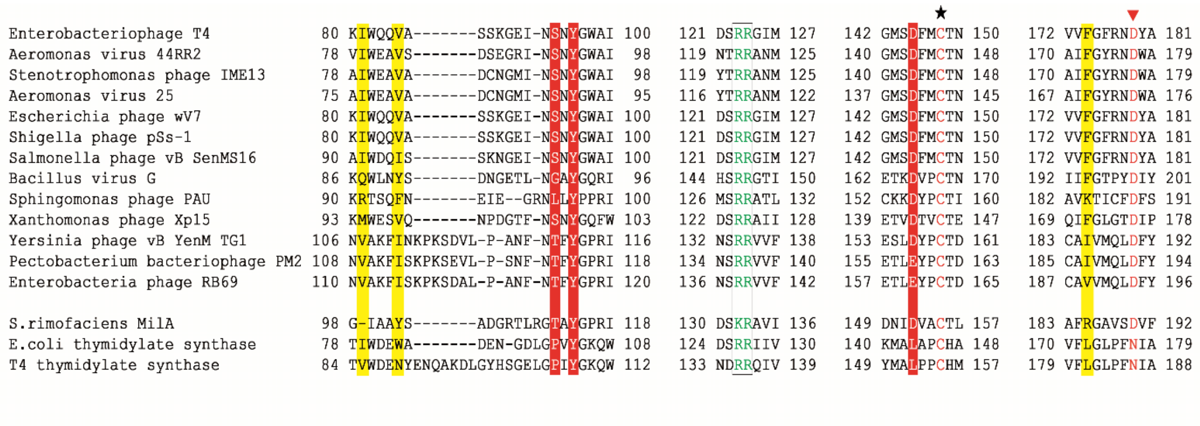

Figure S11 Multiple sequence alignment of putative dCHs and the well-characterized T4dCH, CH (SrMilA), and TS (*E. coli* and T4) proteins. GenBank accession numbers of dCH homologs are as follows: Enterobacteriophage T4 (T4dCH), NP_049659.1; Aeromonas virus 44RR2, NP_932389.1; Stenotrophomonas phage IME1, YP_009217483.1; Aeromonas virus 25, ABF72593.1; Escherichia phage wV7, YP_007004786.1; Shigella phage pSs-1, YP_009110863.1; Salmonella phage vB SenMS16, YP_007501078.1; Bacillus virus G, YP_009015622.1; Sphingomonas phage PAU, YP_007006789.1; Xanthomonas phage Xp15, YP_239304.1; Yersinia phage vB YenM TG1, YP_009200310.1; Pectobacterium bacteriophage PM2, YP_009211464.1; Enterobacteriophage RB69, NP_861738.1; S. rimofaciens MilA (SrMilA), ACA14348.1; *E. coli* thymidylate synthase (EcTS), KXH01147.1; Enterobacteriophage T4 thymidylate synthase (T4TS), NP_049848.1. The key catalytic cysteine residues are indicated with a black star, and the specificity-determining aspartate residues for recognizing the cytosine base are indicated with an inverted, inverted triangle. The phosphate-binding arginine residues are colored green and boxed. Hydrophobic gating residues upon THF-binding are indicated by filled yellow boxes. The signature residues in the dCH family are shaded with filled red boxes.
